# Immunomodulation by durvalumab and pomalidomide in patients with relapsed/refractory multiple myeloma

**DOI:** 10.1038/s41598-021-95902-x

**Published:** 2021-08-12

**Authors:** Mary H. Young, Greg Pietz, Elizabeth Whalen, Wilbert Copeland, Ethan Thompson, Brian A. Fox, Kathryn J. Newhall

**Affiliations:** Bristol Myers Squibb, 3401 Princeton Pike, Princeton, NJ 08648 USA

**Keywords:** Biomarkers, Oncology

## Abstract

This study sought to understand how the programmed death ligand 1 (PD-L1) inhibitor durvalumab and the immunomodulatory agent pomalidomide regulate immune cell activation and function in patients with relapsed/refractory (RR) multiple myeloma (MM). Immunologic changes in peripheral blood and bone marrow of patients treated with durvalumab as monotherapy or in combination with pomalidomide with/without dexamethasone were characterized by assessing subsets of immune cells and gene signatures to understand the immunomodulatory effect of the treatment. Soluble PD-L1 levels were elevated at screening in patients with RRMM but did not correlate with response to durvalumab combination therapy. Immune cell subsets were increased in peripheral blood during treatment with durvalumab and pomalidomide, and combination therapy induced significant gene expression changes in the MM tumor microenvironment versus durvalumab alone. Estimation of cell populations based on RNA sequencing data revealed increased monocytes, neutrophils, and natural killer cells with the combination therapy, but not with durvalumab alone. Additionally, multiplex immunofluorescence of bone marrow demonstrated that immune populations were different in responders versus nonresponders to durvalumab plus pomalidomide with dexamethasone therapy. Overall, durvalumab effectively blocked soluble PD-L1; however, durvalumab monotherapy was not associated with immunologic changes, which were observed with combination therapy.

## Introduction

Multiple myeloma (MM) is a chronic cancer of plasma cells in the bone marrow^1^. Although there are effective therapeutic combinations, patients with MM often relapse and become refractory to standard-of-care treatments such as immunomodulatory imide (IMiD) agents, proteasome inhibitors, and anti-CD38 monoclonal antibodies (e.g., daratumumab), resulting in a worsening prognosis^2,3^. Studies have shown that in MM, there is dysregulation of the immune compartment in the bone marrow, including changes in major cell populations such as natural killer (NK) cells, CD4 + and CD8 + T cells, and dendritic cells, and increases in immune suppressive cell populations, including regulatory T cells (T_reg_), tumor-associated macrophages (TAMs), and myeloid-derived suppressor cells (MDSCs)^4–8^. It has been demonstrated that programmed cell death protein-1 (PD-1) expression is upregulated on T cells isolated from patients with MM, suggesting that this pathway is of importance in mediating the immunosuppressive state in this patient population^9^. Furthermore, studies have shown that while programmed death ligand 1 (PD-L1) is absent from normal plasma cells, it is expressed in MM cell lines and primary MM tumor cells from patients^9–13^. Together, this suggests a role for immunotherapy in treatment of MM.

Monoclonal antibodies that block inhibitory receptors have shown significant clinical activity across a variety of tumor types^14–17^. Blockade of immune checkpoints, such as cytotoxic T-lymphocyte-associated antigen 4 (CTLA-4), PD-1, and PD-L1, has shown clinical activity not only in conventionally immune-responsive tumors such as melanoma and renal cell carcinoma, but also in non-small cell lung cancer (NSCLC) and prostate cancer^16–18^. These checkpoint inhibitors (CPIs) work to limit and potentially reverse T-cell exhaustion by blocking the inhibitory signals to create an effective immune response.

Durvalumab is a human immunoglobulin G1 kappa monoclonal antibody CPI targeted against PD-L1 that has been approved for the treatment of patients with stage III NSCLC and as first-line therapy with etoposide and either carboplatin or cisplatin for extensive-stage small cell lung cancer^19–23^. PD-L1 expressed on tumor cells binds to PD-1 on T cells, leading to downregulation of T-cell activity, which allows tumor cells to evade the immune response. Durvalumab binds human PD-L1 with high affinity, blocking its ability to bind PD-1, thus restoring immune activation with downstream effects on cytokine production, proliferation, cell survival, and transcription factors associated with effector T-cell function^19,20^.

IMiD agents, which have been a mainstay of MM treatment (lenalidomide and pomalidomide), have been shown to downregulate expression of PD-L1 on malignant plasma cells in vitro^24^. Given their distinct mechanisms of action, the combination of an IMiD agent with a CPI may provide additive benefit compared with either agent alone. Combination of an IMiD agent with a CPI, such as the anti-PD-1 antibody pidilizumab, has been shown in vitro to result in an enhancement of MM-targeting activity by augmenting the T-cell-mediated immune response^12,24^. Lenalidomide in combination with PD-1/PD-L1-blocking antibodies blocks bone marrow stroma cell-induced MM growth through induction of interferon-γ (IFN-γ) and granzyme-B in T and NK cells, as well as inhibiting MDSC-mediated immune suppression. The current study investigated the immunomodulatory effect of the CPI durvalumab alone or in combination with the IMiD agent pomalidomide with or without dexamethasone in patients with relapsed/refractory MM (RRMM).

## Methods

### Study design, patients, and treatment

Samples were obtained from patients enrolled in the clinical trial MEDI4736-MM-001 (clinicaltrials.gov identifier: NCT02616640; registered 30/11/15). This was a multicenter, open-label, phase 1b study designed to determine the recommended dose and regimen of durvalumab either as monotherapy or in combination with pomalidomide with or without low-dose dexamethasone in patients with RRMM. Patients must have received at least 2 prior lines of antimyeloma therapy, including lenalidomide and a proteasome inhibitor, and demonstrated disease progression on or within 60 days of completion of the last therapy. This study was conducted in accordance with the International Council for Harmonisation of Technical Requirements for Registration of Pharmaceuticals for Human Use/Good Clinical Practice, the Declaration of Helsinki, and applicable regulatory requirements. The study protocol was approved by local or independent institutional review boards or ethics committees at participating sites. All patients provided written informed consent.

Patients were randomized to 1 of 3 treatment arms. In the durvalumab monotherapy arm (Arm A), patients received 1500 mg intravenous (IV) durvalumab on day 1 of each 28-day cycle. Patients randomized to Arm B received durvalumab as in Arm A plus oral pomalidomide 4 mg/day on days 1 to 21. Patients in Arm C received oral dexamethasone 40 mg/day (≤ 75 years of age) or 20 mg/day (> 75 years of age) on days 1, 8, 15, and 22 of the 28-day cycle in addition to durvalumab and pomalidomide. At the investigator’s discretion, patients randomized to Arms A or B could switch to Arm C upon progression or lack of efficacy.

Responders were defined as patients with a partial response or better using International Myeloma Working Group Uniform Response Criteria^25^. The focus of this paper is on exploratory endpoints, including pharmacodynamic, mechanistic, and predictive biomarkers of durvalumab, both as a single agent and in combination with pomalidomide with or without dexamethasone.

### Soluble PD-L1 (sPD-L1) analysis

Blood was collected at screening in serum separator tubes, inverted, and allowed to stand at room temperature (RT) for 60 to 120 min. Tubes were centrifuged at 1500 *g* for 10 min at RT, and serum was transferred to a vial and stored at − 70 °C. Serum samples for electrochemiluminescence assays were shipped frozen to the central laboratory (Laboratoires Cerba, Saint Ouen l’Aumône, France), and to the analytical laboratory (Intertek, London, UK) where the validated quantitative sPD-L1 electrochemiluminescence assay was performed. The upper and lower limits of detection were 1000 pg/mL and 15.6 pg/mL, respectively. Analyses provided screening sPD-L1 values for each evaluable patient sample and were compared with samples from healthy volunteers. The healthy serum samples were acquired through Intertek (San Diego, CA); all samples from these volunteers were consented.

### Flow cytometric analyses of peripheral blood

Peripheral blood was obtained at screening, cycle 1 day 1 (C1D1; pretreatment), cycle 1 day 8 (C1D8), cycle 1 day 15 (C1D15), cycle 2 day 1 (C2D1), and cycle 2 day 15 (C2D15). Whole blood samples for immunophenotyping were collected in sodium-heparin tubes and shipped same-day at ambient temperature directly to the analytical laboratory (Quintiles: Marietta, GA or Livingston, Scotland). Cells were resuspended and stained with 6 separate panels using antibody-fluorophore conjugates (BD Biosciences, San Jose, CA) on a BD FACSCanto II (8 color, 3 laser) instrument (see Supplementary Table for a full listing of 6 panels with fluorophores and antibody clones used). Flow cytometry was performed at Quintiles Laboratories (Marietta, GA, or Livingston, Scotland), and data were analyzed using FACSDiva software (BD Biosciences). Analysis included evaluation of immune cell subsets including T cells, B cells, NK cells, MDSCs, and T_reg_s. Ki67 staining was used to monitor proliferation of T cells and NK cells. Additional panels provided the evaluation of suppressor cells, checkpoint molecule expression, T-cell activation, and T-cell exhaustion. Patient samples were evaluated longitudinally through C2D15, and reported data included percentage of population, absolute cell number, and mean fluorescence intensity as appropriate.

### RNA sequencing of bone marrow

RNA from bone marrow samples was obtained from patients at screening and after 6 weeks of treatment (at the C2D15 visit) using tubes with either dimethylsulfoxide (DMSO) or TRIzol to stabilize the material. RNA sequencing was performed at EA Genomics (Q^2^ Solutions, Morrisville, NC) using the Qiagen Micro RNeasy kit (Hilden, Germany). Quality control checks included spectrophotometric measurements and agarose gel analysis. RNA sequencing libraries were prepared using polyA enrichment and strand-specific library construction using barcodes. Samples were sequenced on an Illumina HiSeq 2500 with 2 × 50 bp read lengths using TruSeq SBS v4 chemistry (Illumina, San Diego, CA).

RNA-sequencing count data were normalized using trimmed mean of M values (TMM) and then transformed to log_2_ counts per million along with observation level weights using voomWithQualityWeights from the limma R package^26^. Genes with a TMM count of at least 1 in 10% of libraries, resulting in approximately 19,000 genes, were retained for further analysis. No libraries were removed because of poor quality control. To find differentially expressed genes between screening and C2D15, a linear model with a random effect for individual was run within each cohort. To study cell type proportion changes, cell markers were used from the xCell signature gene sets (University of San Francisco Institute of Computational Health Sciences), which are based on pure cell types from multiple sources^27^. With the xCell gene sets, competitive gene set analysis was performed between screening and C2D15 using the camera function from the limma R package. Finally, visualization of the results was done with gene set variation analysis (GSVA) to calculate a summary metric for each gene set. The *P* value and adjusted *P* value threshold were calculated using limma (R package). Linear models included the visit and the bone marrow mononuclear cell storage condition (TRIzol vs. DMSO), plus a blocking term (with duplicate correlation calculated) for patients who had samples matched between screening and C2D15.

### T-cell receptor analysis

T-cell receptor (TCR) beta chain sequencing was performed using the immunoSEQ platform (Adaptive Biotechnologies, Seattle, WA). Bone marrow mononuclear cell (BMMC) samples with 400,000 to 5 million cells were frozen (n = 74; 32 matched patients between screening and cycle 2). Adaptive Biotechnologies performed the DNA extraction, TCR beta amplification, and sequencing. The immunoSEQ Analyzer 3.0 software was used to calculate the clonality for each sample and the pairwise comparisons between screening and cycle 2. Clonality is the inverse of the normalized Shannon entropy such that values near 1 represent samples with many copies of only a few clones, while values near zero are highly polyclonal.

### Multiplex immunofluorescence analysis

Bone marrow biopsies for multiplex immunofluorescence (mIF) were isolated according to standard institutional procedures. Tissue was fixed in 10% formalin, transferred to 70% ethanol, and shipped at ambient temperature to the central laboratory. Two panels were used to characterize the cellular content of the bone marrow biopsy samples using mIF with a PerkinElmer Vectra instrument (PerkinElmer, Waltham, MA). Visiopharm was used to calculate the cell marker densities based on the hematoxylin and eosin-stained nuclei and marker intensities. The innate immune panel had antibodies for CD138, CD38, PDL1, CD163, CMAF, and CD11c. The lymphocyte panel had antibodies for CD3, CD8, CD20, PD1, FOXP3, and CD138. Cell densities for pairs of markers were used to look at more specific populations. On the tumor/myeloid panel, these pairs were used: CD38/CD138, CD138/PDL1, CD38/PDL1, CD163/CMAF, CD163/PDL1, CD11c/PDL1, and CMAF/PDL1. On the lymphocyte panel, these pairwise densities were calculated: CD3/CD8, CD3/FOXP3, CD3/PD1, and CD8/PD1. Each panel was applied on a different slide; cell densities from each slide were analyzed independently. The comparison between responders and nonresponders was done using a Wilcoxon rank sum test using GraphPad Prism (GraphPad Software, San Diego, CA).

### Ethical approval

The study was approved by the following institutional review boards:

University of Arkansas for Medical Sciences Institutional Review Board, Slot 636 4301 W Markham Street, Little Rock, AR 72205 USA.

John Hopkins Medical Institutions IRB (JHMIRB), 1620 McElberry Street, Baltimore, MD 21205 USA.

Western Institutional Review Board (WIRB), 1019 39th Avenue SE, Suite 120, Puyallup, WA 98341 USA.

Weil Cornell Medical College Institutional Review Board, 1300 York Avenue, Box 89, New York, NY 10065 USA.

Advarra IRB, 6940 Columbia Gateway Drive, Suite 110, Columbia, MD 21046 USA.

Dana-Farber Cancer Institute Institutional Review Board, 450 Brookline Avenue, Boston, MA 02215 USA.

Biomedical Research Alliance of New York (Brandy), 1981 Marcus Avenue, Suite 210, Lake Success, NY 11042 USA.

Cleveland Clinic IRB, 9500 Euclid Ave OS-1, Cleveland, OH 44195 USA.

Comité de Protection des Personnes Nord Ouest IV, Bâtiment ex USN B (RDC) 6 rue du Pr. Laguesse, Lille Cedex 59037, France.

Medisch Ethische Toestings Commissie Erasmus MC, Dr. Molewaterplein 40, Kamer AE-337, Rotterdam 3015 GD, Netherlands.

Health Research Ethics Board of Alberta, 103 Ave NE, Suite 1500-10104, Edmonton T5J4A7, Alberta, Canada.

Ethik-Kommission an der Medizinischen Fakultät der Eberhard-Karls-Universität und am Universitätsklinikum Tübingen, Gartenstraße 47, 72074 Tübingen, Germany.

Comitato Etico Interaziendale, A.O Città della Salute e della Scienza di Torino, C.so Bramante 88/90, Torino 10126, Italy.

Comitato Etico Fondazione IRCCS- Instituto Nazionale dei Tumori Di Milani, Via Venzian 1, Milano 20133, Italy.

Comitato di Bioetica della Fondazione I.R.C.C.S Policlinico San Matteo, Viale Golgi 19, Pavia 27100, Italy.

Comitato Etico IRCCS Istituto Nazionale per lo Studio e la Cura dei tumori, Via Mariano Semmola, Napoli 80131, Italy.

Comitato Etico Indipendente Istituto Clinico Humanitas – IRCCS, Via Manzoni 56, Rossano, Milano 20089, Italy.

Comite Etico de Investigacion Clinica de Navarra, Irunlarrea 3, Pamplona 31008, Spain.

## Results

### Soluble PD-L1 (sPD-L1) levels

**Figure 1:**
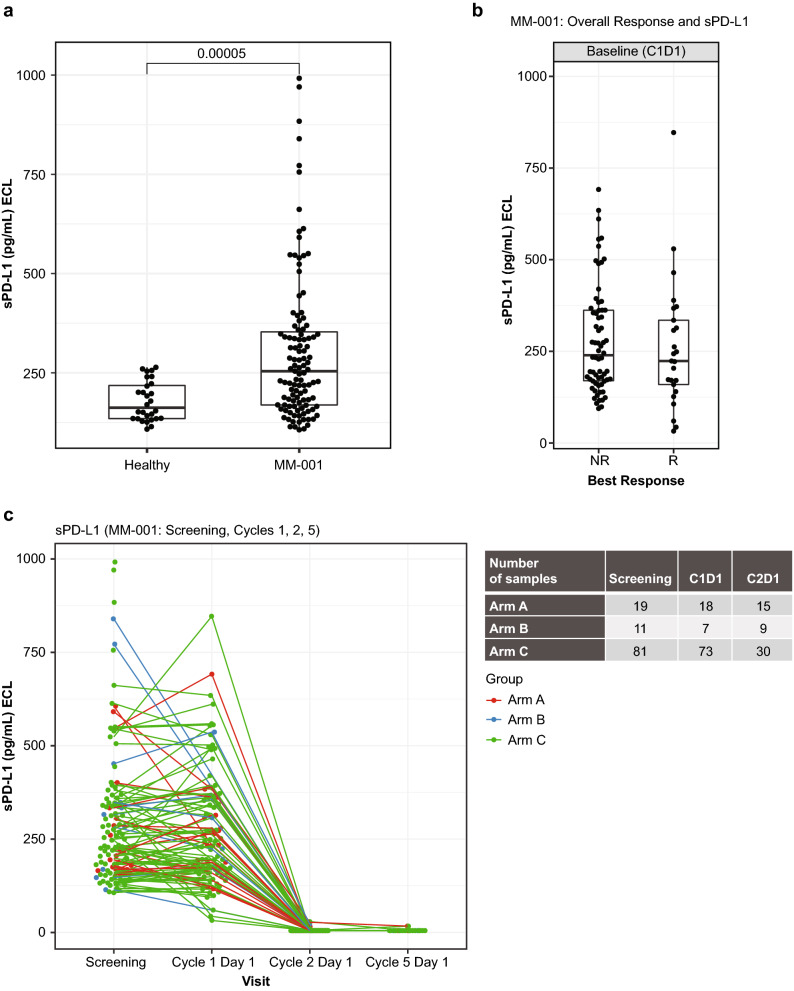
sPD-L1 concentration measured using a quantitative electrochemiluminescence assay: (**a**) sPD-L1 is significantly elevated in RR MM compared with healthy volunteers (*P* = 0.0005); (**b**) sPD-L1 at screening does not correlate with response to durvalumab; R, n = 25; NR, n = 66; (**c**) sPD-L1 levels were below the limit of quantitation after treatment with durvalumab. C1D1, cycle 1 day 1; C2D1, cycle 2 day 1; ECL, enhanced chemiluminescent; NR, nonresponders; R, responders; RR MM, relapsed refractory multiple myeloma; sPD-L1, Soluble PD-L1.

At the C1D1 timepoint, sPD-L1 was measured in 18 patients in the durvalumab monotherapy arm (Arm A), 7 patients in the durvalumab + pomalidomide arm (Arm B), and 73 in the durvalumab + pomalidomide + dexamethasone arm (Arm C). The results demonstrated that sPD-L1 is significantly elevated in patients with RRMM compared with 28 healthy volunteers of a similar age (*P* = 0.00005; Fig. 1a). However, there was no association of sPD-L1 between screening (C1D1) and response to therapy (Fig. 1b). There was also no association of sPD-L1 levels when comparing patients with high-risk cytogenetics versus other patients. Following durvalumab treatment, sPD-L1 levels were below the limit of quantitation in all patients, indicating complete target coverage, even in the presence of elevated screening levels of sPD-L1 (Fig. 1c).

### Immune cell subsets in peripheral blood

**Figure 2:**
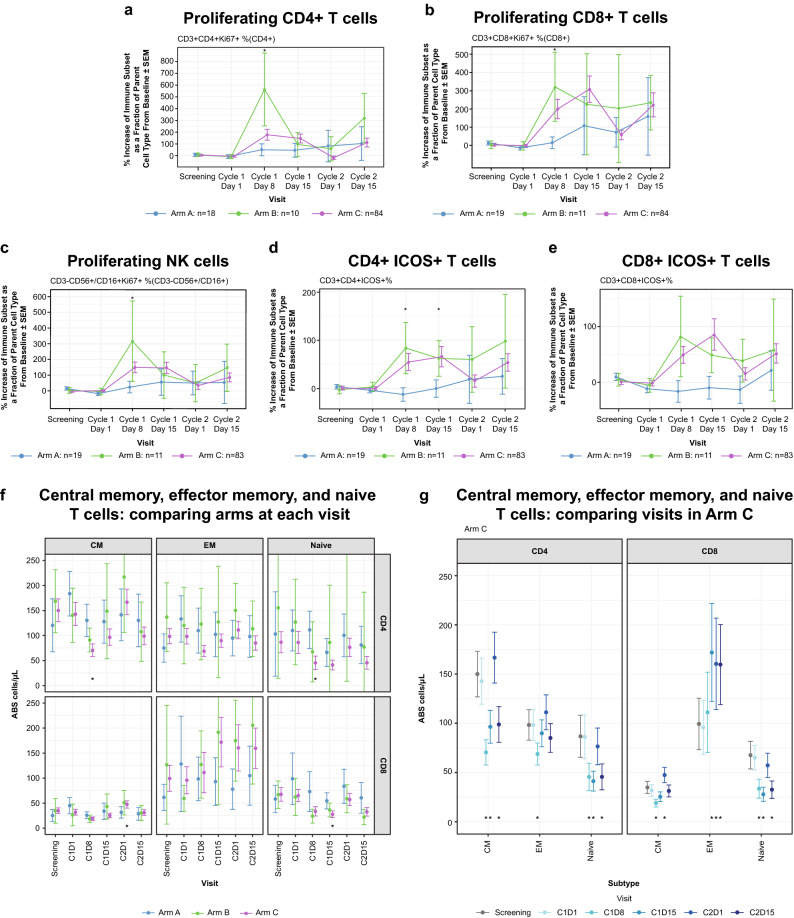
Changes in immune cell subsets in peripheral blood using flow cytometry during treatment with durvalumab monotherapy (Arm A), durvalumab + pomalidomide (Arm B), or durvalumab + pomalidomide + dexamethasone (Arm C). (**a**) CD4 T-cell proliferation; (**b**) CD8 T-cell proliferation; (**c**) NK-cell proliferation; (**d**) CD4 + ICOS + ; (**e**) CD8 + ICOS + ; (**f**) and (**g**) central memory (CM), effector memory (EM), and naive T cells: comparing arms at each visit (**f**) and comparing visits in Arm C (**g**). Asterisks in a-e indicate that Arm C has a nonoverlapping SEM intervals with Arm A and/or Arm B. Asterisks in f show where there is a non-overlapping interval in Arm C versus Arm A. Asterisks in g show which visits are changed (nonoverlapping interval) from the baseline (screening/C1D1) visits. ABS, antibodies; CM, central memory; EM, effector memory; ICOS, inducible T-cell co-stimulator; NK, natural killer; POM, pomalidomide; SEM, standard error of the mean.

Results of flow cytometry in the peripheral blood conducted at screening, C1D1, C1D8, C1D15, C2D1, and C2D15 are summarized by arm in Fig. 2, with up to 19, 11, and 84 samples analyzed at each visit in arms A, B, and C, respectively. There was a 50% increase in the proportion of proliferating (Ki67 +) CD4 T cells following durvalumab monotherapy (Fig. 2a). In patients treated with pomalidomide in Arm B or Arm C, larger increases (150–500%) in the proportion of proliferating CD4 T cells, CD8 T cells, and NK cells were detected as early as C1D8 (Fig. 2a–c), and for Arm C this was followed by a decline at the start of cycle 2 (prior to dosing) and then an increase again by C2D15. Both cohorts that received pomalidomide in combination with durvalumab had additional increases in T_reg_s (CD4 + FoxP3 + CD127 +) (not shown) and CD4 + ICOS + helper T-cell subsets (Fig. 2d) as a proportion of CD4 cells. In the CD8 + compartment of Arm C patients, there were increases in CD8 + ICOS + (Fig. 2e) subsets and effector memory (Fig. 2f,g), and at C1D8 or C1D15, while the central memory cells had a slight decrease initially (C1D8) but then rebounded to higher than initial levels at the start of cycle 2. Some T-cell subsets had a decrease in Arm C patients: there was a 50% decrease in the absolute counts of naive CD8 + T cells and both central memory and naive CD4 + T cells were also decreased by approximately 50% on treatment (Fig. 2f,g). These decreases were not seen in Arm A patients, and Arm B patients trended similarly, but have wider confidence intervals due to the lower number of patients compared with Arm C.

### Estimation of cell populations based on RNA sequencing data

**Figure 3:**
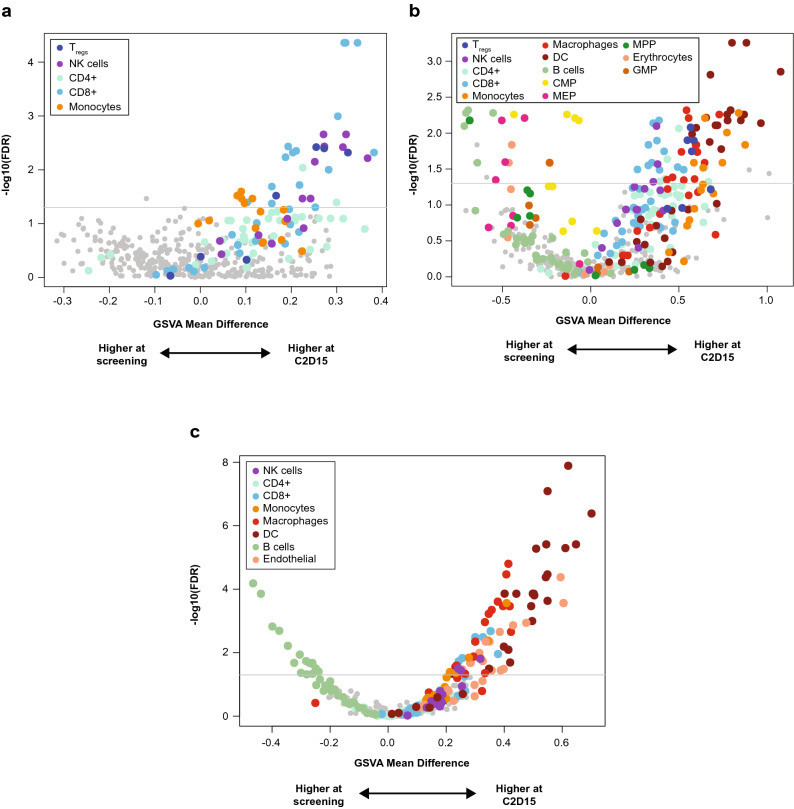
Estimation of immune cell populations based on RNA sequencing data using the gene set enrichment analysis using gene sets from xCell ^27^ at screening and cycle 2 day 15 (C2D15) treated with: (**a**) durvalumab monotherapy (screening n = 15; C2D15 n = 7); (**b**) durvalumab + pomalidomide (screening n = 6; C2D15 n = 2); (**c**) durvalumab + pomalidomide + dexamethasone (screening n = 53; C2D15 n = 50). The y-axis is the negative log_10_ of the *P* value.

Whole bone marrow aspirate samples from patients in all arms were analyzed by RNA sequencing at screening and C2D15. We estimated the relative abundance of different cell populations in each of the 3 arms by applying gene set enrichment analysis of immune cell types based on gene sets from xCell (Fig. 3)^27^ and comparing C2D15 to screening samples. Following durvalumab monotherapy, there were inferred increases in CD8 + T-cell, NK-cell, and T_reg_ genes (Fig. 3a). In the combination arms with pomalidomide (Arms B and C; Fig. 3b,c), an increase in genes from dendritic cells, monocytes, and macrophages, in addition to CD8 + T cells and T_reg_s, was detected. In addition, in Arms B and C, many of the B-cell-related gene sets were enriched in the screening relative to C2D15 samples, consistent with a decrease in tumor plasma cells on treatment.

### Gene expression changes in the MM tumor microenvironment

**Figure 4:**
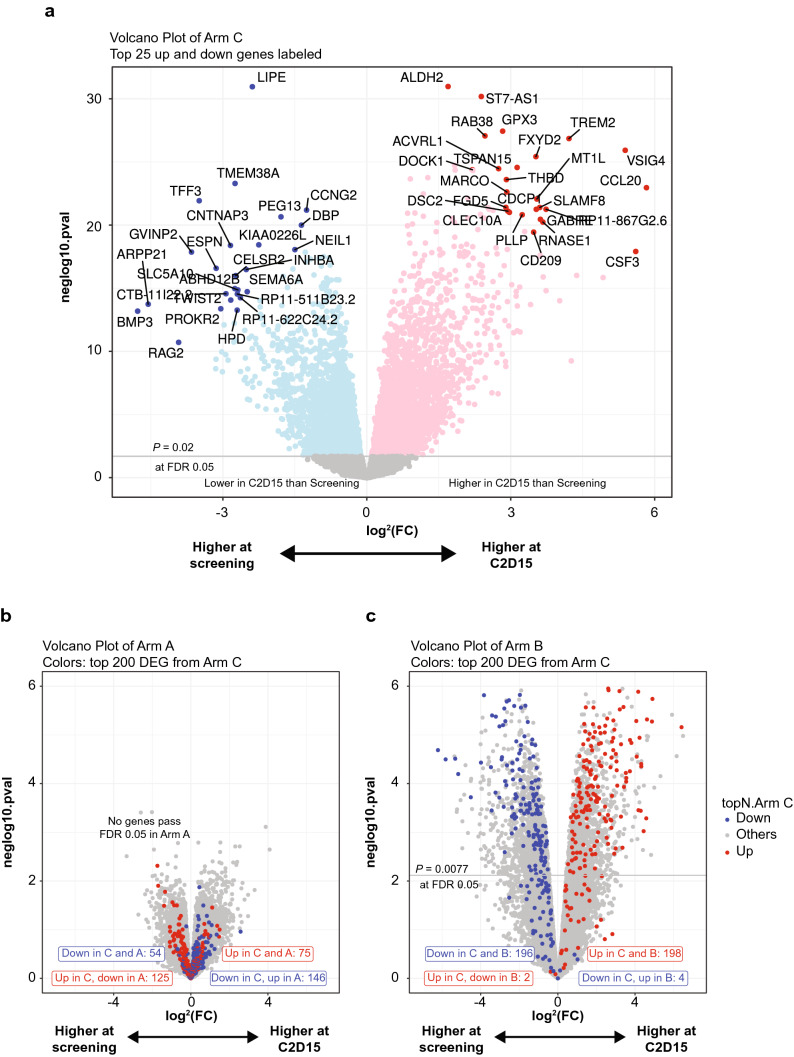
Difference in transcriptional profile of whole bone marrow samples at screening and cycle 2 day 15 (C2D15) from patients in all three cohorts using RNA sequencing. In panel A, the volcano plot shows each gene as a point and its log^2^ fold change (x-axis) between screening and C2D15 for patients in Arm C (durvalumab, pomalidomide, dexamethasone), where positive values mean higher expression of the gene in C2D15 compared to screening, and negative values are the inverse; and, the y-axis shows the negative log_10_ of the raw *P* value with a horizontal line drawn where the multiple-testing adjusted *P* value (False Discovery Rate – FDR) is at 0.05. In Arm C there were 50 samples at C2D15 and 53 samples at screening. In B and C, the volcano plots are based on the differences between C2D15 and screening for Arm A and Arm B, respectively. In those panels, the top 200 differentially expressed genes from Arm C are colored red if they were upregulated on treatment in Arm C and blue if they were downregulated on treatment in Arm C so that it could be determined if they were changing in the same direction in those smaller cohorts. In Arm A (durvalumab), there were 15 patients at screening and 7 patients at C2D15. In Arm B (durvalumab + pomalidomide), there were 6 patients at screening and 2 patients at C2D15. C2D15, cycle 2 day 15; FDR, false discovery rate.

We used the RNA sequencing data to look for individual transcripts that changed within the first 2 months of treatment. Figure 4a shows a volcano plot of the top differentially expressed genes in the durvalumab + pomalidomide + dexamethasone arm (Arm C) between screening and C2D15 such that genes which have higher expression at C2D15 compared with screening have positive fold changes along the x-axis. Figure 4b,c show the screening versus C2D15 volcano plots for Arms A and B, with the top 200 genes from Arm C highlighted in red and blue. Durvalumab alone did little to change the transcriptional profile of bone marrow, as there are no individual genes that have adjusted *P* values lower than 0.05, and the differential genes from Arm C are not consistently changed in the direction of their fold change (Fig. 4b). The addition of pomalidomide increased the number of differential genes on treatment in Arms B and C (Fig. 4a,c), and the specific gene expression changes on treatment in Arm B were highly consistent with those seen in Arm C (Fig. 3c), despite only 2 samples at C2D15 in Arm B. In Arms B and C, the chemoattractants IL1B, CXCL1, and CXCL9 were upregulated on C2D15, as well as other inflammatory factors such as TNF, PD-L1 (CD274), and PD-1 (PDCD1). Downregulated genes included those from B cells, CD19, CD79B, and BLK. Furthermore, CXCR3, Tim-3 (HAVCR2), and IL7 were also increased, suggesting a role for T-cell function. Separating the Arm C patients by responders and nonresponders did not reveal any significant gene differences at screening or on treatment (not shown).

### IFN-γ signature

**Figure 5:**
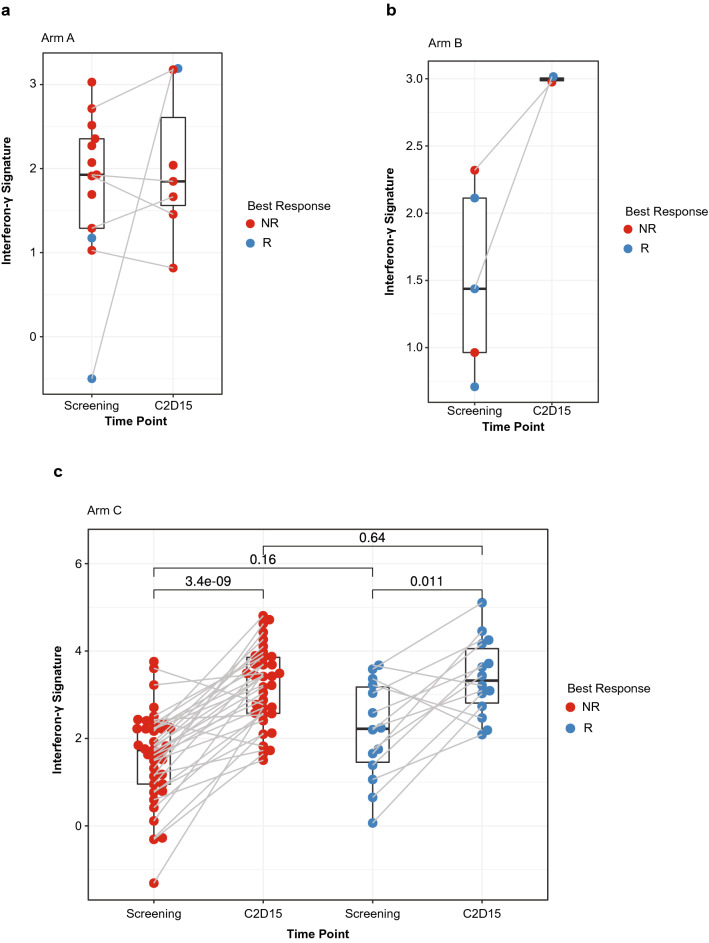
Normalized IFN-γ 4-gene signature in bone marrow samples of responders and nonresponders at screening and C2D15 treated with: (**a**) durvalumab monotherapy (screening n = 13; C2D15 n = 7); (**b**) durvalumab + pomalidomide (screening n = 5; C2D15 n = 2); (**c**) durvalumab + pomalidomide + dexamethasone (screening n = 51; C2D15 n = 49). Patients without a best response are excluded. C2D15, cycle 2 day 15; IFN, interferon; NR, nonresponder; R, responder.

Previous studies have used an IFN-γ-based 4-gene signature as a predictive biomarker of efficacy in NSCLC^28^. To determine if this gene signature was induced in any of the patients in this study upon treatment, we used the RNA sequencing data to compare screening and C2D15 bone marrow samples that were also separated into nonresponder and responder groups. There were insufficient samples from Arms A and B to draw inferences about the effect of treatment on the IFN-γ-based gene signature (Fig. 5a,b). However, in Arm C, the increased numbers of samples allowed us to divide the patients by response, with the signature score significantly upregulated in both responder and nonresponder populations (Fig. 5c). Although the IFN-γ-based gene signature increased on treatment, we found that the level at screening was not associated with treatment response.

### T-cell receptor sequencing

**Figure 6:**
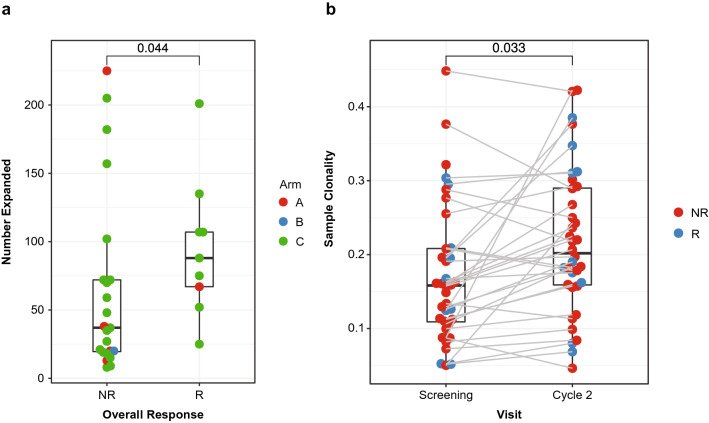
TCR clone expansion and clonality. (**a**) Quantification of the number of expanded clones in patient samples for 9 responders (R) and 23 nonresponders (NR). Each point is a patient, and the y-axis shows the number of T-cell clones that significantly expanded from screening to C2D15 sample collection in the BMMCs. (**b**) Clonality of templates at screening and C2D15 in combined responders and nonresponders. Gray lines connect patients who have both a screening and cycle 2 sample (n = 33). There are 36 samples at screening and 36 samples at cycle 2. For both (**a**) and (**b**), the *P* value is from a Wilcoxon rank test of the samples in the two groups, where the patient pairing is ignored for (**b**). BMMC, bone marrow mononuclear cells; C2D15, cycle 2 day 15; *TCR* T-cell receptor.

TCR beta chain sequences were determined in BMMC samples from patients at screening and at C2D15. For each of the 32 patients who had data for screening and cycle 2, the TCR beta sequences were compared and each clone was determined to be increasing, decreasing, unchanged, or too low to determine. Patients who responded to therapy had a higher number of clones that expanded on treatment compared with nonresponders (*P* = 0.044; Fig. 6a). Additionally, the clonality was increased overall on treatment (*P* = 0.032; Fig. 6b).

### Immunofluorescence of bone marrow

**Figure 7:**
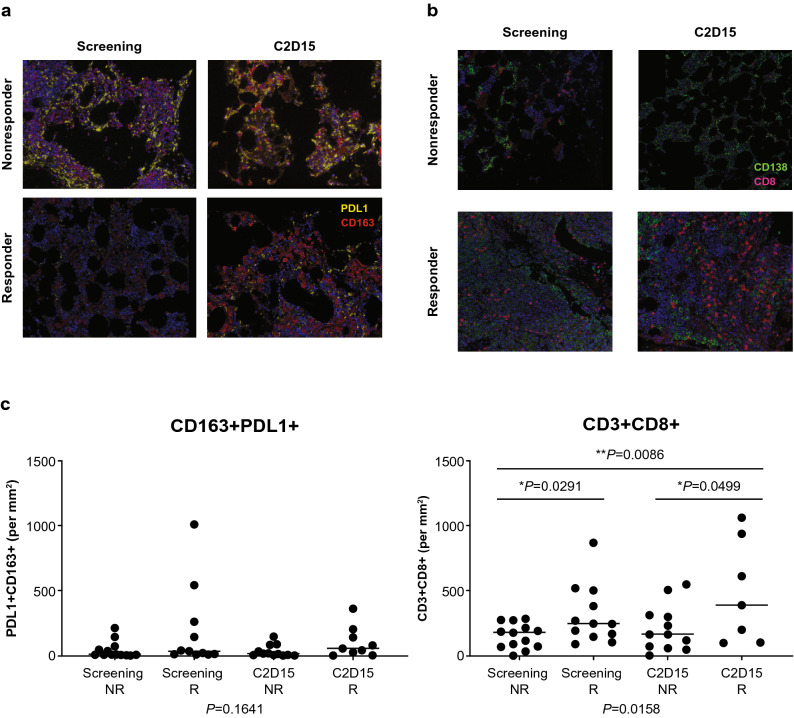
Immunofluorescence staining of bone marrow biopsies from responders and nonresponders in the durvalumab + pomalidomide + dexamethasone arm at screening and cycle 2 day 15 (C2D15). Results from the durvalumab monotherapy and durvalumab + pomalidomide arms were not evaluable. (**a**) PD-L1 and CD163 fluorescence; (**b**) CD8 and CD138 fluorescence. (**c**) Enumeration of positive cells from A and B. Significance was determined by one-way ANOVA and unpaired t test. ANOVA, analysis of variance.

We examined immunofluorescence staining of bone marrow biopsies using 2 panels: one that focused on the innate immune infiltrates and another that was used to interrogate the lymphocyte compartment, including T-cell checkpoint expression. For this analysis, only the durvalumab + pomalidomide + dexamethasone group (Arm C) was evaluable. Each response group was assessed with both panels at screening and on C2D15 (Fig. 7a,b). Potentially due to the low sample numbers, there were few significant differences between responders and nonresponders at screening or C2D15. Responders appeared to have a slight increase in CD163 + PDL1 + cells at both screening and C2D15, although this was not significant (*P* = 0.164). There were no other differences in the measured cell densities between responders and nonresponders, including no difference for PD-L1 + tumor cells. There were significant changes in the T-cell infiltrates of tumors: responders had an increase in CD8 + T cells at screening and C2D15 (*P* = 0.0158), including those that also expressed PD-1 + (*P* = 0.020) (Fig. 7c). There was also an increase in CD3 + PD1 + T cells as a whole (*P* = 0.015) at C2D15 in the responders. Although not significant, there also appeared to be a slight increase in Foxp3 + T cells in responders at screening and C2D15 (*P* = 0.100). Taken together, these results suggest that treatment with durvalumab, pomalidomide, and dexamethasone induced a more robust T-cell response in patients that responded to treatment as opposed to those who did not.

## Discussion

CPIs have led to positive outcomes in several solid tumor types^16–18,21–23^. However, recent trials with the anti-PD-1 pembrolizumab in combination with an IMiD agent and dexamethasone in MM were stopped due to decreased survival and increased adverse events^29,30^; subsequently, all studies with CPIs and IMiD agents were temporarily stopped based on these findings. The future use of CPIs in hematologic malignancies is dependent on a complete understanding of the immunologic impact of these agents in this setting.

The current study was designed to investigate the immunomodulatory effects of checkpoint blockade with durvalumab alone and in combination with pomalidomide and dexamethasone. Serum sPD-L1 shed from the surface of several immune lineages, including plasmacytoid dendritic cells, macrophages, and MDSCs, has been shown to be a predictive and prognostic biomarker in various human cancers including MM, diffuse large B-cell lymphoma, NSCLC, and hepatocellular carcinoma^31,32^. In MM, the overall response rate to older therapies, including proteasome inhibitors, has been shown to be higher in patients with low sPD-L1 expression than in those with high sPD-L1 expression^33^. Here, we confirmed previous reports of elevated sPD-L1 in patients with RRMM compared with age-matched healthy volunteers. Durvalumab binds to circulating sPD-L1, which may limit exposure of tumor cells to the drug. As a result, sPD-L1 has also been used as a biomarker of target coverage in solid tumor trials of durvalumab, where treatment has resulted in dose-dependent suppression of free sPD-L1^34,35^. In contrast with these previous trials, in this study sPD-L1 levels were below the limit of quantitation following durvalumab treatment, indicating complete target coverage, even in the presence of elevated screening levels of sPD-L1 in patients with MM.

Similar to the known pharmacodynamic effects of durvalumab monotherapy in solid tumors, we saw modest effects of durvalumab on T- and NK-cell proliferation in peripheral blood, which was further increased with addition of pomalidomide. Of interest, the addition of dexamethasone to the durvalumab + pomalidomide combination did not inhibit the immune response; instead, the onset of immune cell activation was delayed by about 7 days and was not sustained.

A number of studies have reported correlation between various molecular markers, including expression level of PD-L1 in tumor and immune cells and IFN-γ gene expression signatures, and the clinical activity of CPIs, including durvalumab^17,36^. Experience with durvalumab in solid tumors showed that greater responses were observed in patients with PD-L1-positive tumors, with much lower rates of response in patients with PD-L1-negative tumors^37^. In addition, tumors that had an elevated IFN-γ 4-gene signature (IFN-γ, CD274, LAG3, and CXCL9) responded better to treatment^28^. In the study reported here, there was an increase of this signature on treatment in Arm C; there were insufficient data to draw conclusions in the other arms. Notably, the magnitude of the IFN-γ-based 4-gene signature at screening was not associated with treatment response to durvalumab combination therapy in RRMM.

Pomalidomide has shown efficacy in combination with dexamethasone for patients with RRMM who experienced relapse on prior lenalidomide and bortezomib^38,39^. Here, in the triplet regimen arm, the IFN-γ 4-gene signature was significantly upregulated in both responder and nonresponder populations following treatment. The changes in gene signatures observed during treatment with pomalidomide resembled those that have been observed with IMiD agent treatment alone in other hematologic malignancies, and are indicative of the strong immunomodulatory effects of IMiD agents^40^. As a result, this study provided us with additional insight into the mechanism of action of IMiD agents in the MM tumor microenvironment. Pomalidomide also had a clear immunostimulatory effect, increasing cycling T-cell and NK-cell populations (including T_reg_, helper T-cell, effector memory cell, and central memory cell subsets), dendritic cells, monocytes, and macrophages, as well as an IFN-γ signature, similar to previous studies with other IMiD agents^41–45^. The addition of pomalidomide likely increased the expression of these genes, owing to increased T-cell activation in these samples. This is consistent with our earlier work that observed increased activated CD4 + and CD8 + T cells in bone marrow in patients treated with pomalidomide compared with treatment-naive patients^46^. Because all arms in this study included durvalumab, it was not possible to determine whether its addition to pomalidomide had any differential effect.

TCR beta chain sequencing of bone marrow samples showed that there was an overall expansion in T-cell clonality in patients receiving treatment. Furthermore, responders had more individual clonal expansion than nonresponders. While this is consistent with observations in patients with solid tumors treated with checkpoint inhibitors^47^, it is not clear whether this was due to durvalumab since all patients in the study were treated with the drug. The combination of durvalumab and pomalidomide induced significant inflammatory changes in the blood and bone marrow, whereas there was little evidence for immunostimulatory activity with durvalumab monotherapy. The apparent lack of immunomodulation with durvalumab when given in combination with pomalidomide in MM may further explain the limited clinical efficacy of these agents in this setting.

A more complete understanding of the clinical benefit of immuno-oncology agents in this setting due to enhanced immune cell competency will inform optimal therapeutic sequencing and use of rational combination regimens. An understanding of the interplay of the distinct mechanisms of cereblon E3 ligase modulators, chimeric antigen receptor T cells, and T-cell engagers will be key to development of the next generation of combination therapies for RRMM.

## Supplementary Information

Supplementary Table.

## Data Availability

BMS policy on data sharing may be found at https://www.bms.com/researchers-and-partners/independent-research/data-sharing-request-process.html.
