# Supplementary material for: Immunomodulation by durvalumab and pomalidomide in patients with relapsed/refractory multiple myeloma
Source: Sci Rep. 2021 Aug 12;11:16460. doi: 10.1038/s41598-021-95902-x (PMC8361181; doi:10.1038/s41598-021-95902-x)
Supplement: Supplementary file 1 — Supplementary Table. [file 41598_2021_95902_MOESM1_ESM.pdf]

Immunomodulation by durvalumab and pomalidomide in patients with relapsed/refractory multiple myeloma

Mary H. Young[1§∞]; Greg Pietz[1∞*]; Elizabeth Whalen[1§]; Wilbert Copeland[1]; Ethan Thompson[1]; Brian A. Fox[1]; Kathryn J. Newhall[1§*]

[1]Bristol Myers Squibb, Princeton, NJ, USA
[§]Employee at the time of the study
[∞]Denotes equal contribution

*Correspondence to:
Greg Pietz
Bristol Myers Squibb
3401 Princeton Pike
Princeton, NJ 08648
Greg.Pietz@bms.com

SUPPLEMENTARY TABLE

Table. Antibody clones used in flow cytometry of peripheral blood.

Panel	Antigen	Clone	Conjugate
Activation	CD4	RPA-T4	PerCP-Cy 5.5
Activation	CD3	SK7	PE-Cy7
Activation	IgG1	MOPC-21	PE
Activation	CD45	HI30	V500
Activation	IgG2a	G155-178	APC
Activation	CD8	SK1	APC-H7
Activation	CD38	HIT2	BV421
Activation	ICOS	DX29	PE
Activation	HLA-DR	L243	APC
Activation	CD45RO	UCHL1	FITC
Activation	CCR7	150503	PE
Activation	CD45RA	HI100	APC
Activation	IgG1	X40	BV421
Activation	CD3	UCHT1	BV421
Checkpoint	CD4	RPA-T4	PerCP-Cy 5.5
Checkpoint	CD3	SK7	PE-Cy7
Checkpoint	IgG1	MOPC-21	PE
Checkpoint	CD45	HI30	BV510
Checkpoint	CD8	SK1	APC-H7
Checkpoint	PD-1	EH12.1	BV421
Checkpoint	LAG-3	3DS223H	PE
Checkpoint	TIM-3	F38-2E2	APC
Checkpoint	IgG1	X40	BV421
MDSC	CD33	WM53	BV421
MDSC	CD19	SJ25C1	BV510
MDSC	CD56	NCAM16.2	BV510
MDSC	CD15	W6D3	FITC
MDSC	CD14	MOP9	PE
MDSC	HLA-DR	G46-6	PerCP-Cy 5.5
MDSC	CD11b	ICRF44	APC
MDSC	CD3	UCHT1	BV510
TBNK	CD19	HIB19	BV421
TBNK	CD3	SK7	FITC
TBNK	CD16	B73.1	PE
TBNK	CD56	NCAM16.2	PE
TBNK	CD45	HI30	PerCP-Cy 5.5
TBNK	CD8	SK1	APC
TBNK	CD14	MOP9	APC-H7
TBNK	CD4	SK3	BV510

TNK Proliferation	CD4	RPA-T4	PerCP-Cy 5.5
TNK Proliferation	CD56	NCAM16.2	BV421
TNK Proliferation	CD45	HI30	V500
TNK Proliferation	CD8	HIT8a	PE
TNK Proliferation	CD7	M.T701	PE-Cy7
TNK Proliferation	CD16	3G8	APC-H7
TNK Proliferation	Ki67	B56	AF488
TNK Proliferation	IgG1	MOPC-21	AF488
TNK Proliferation	CD3	UCHT1	APC
T_{reg}	CD3	SK7	PerCP-Cy 5.5
T_{reg}	CD127	hIL-7R-M21	BV421
T_{reg}	CD45RA	HI100	BV510
T_{reg}	CD25	M-A251	PE
T_{reg}	FoxP3	259D/C7	A647
T_{reg}	IgG1	MOPC-21	A647
T_{reg}	CD4	SK3	FITC

MDSC, myeloid-derived suppressor cell; NK, natural killer; TBNK, T- B- and NK-cells; TNK, T cells expressing T and NK cell markers.
